# The Damage Caused by Decline Disease in Bayberry Plants through Changes in Soil Properties, Rhizosphere Microbial Community Structure and Metabolites

**DOI:** 10.3390/plants10102083

**Published:** 2021-09-30

**Authors:** Haiying Ren, Hongyan Wang, Xingjiang Qi, Zheping Yu, Xiliang Zheng, Shuwen Zhang, Zhenshuo Wang, Muchen Zhang, Temoor Ahmed, Bin Li

**Affiliations:** 1 Institute of Horticulture, Zhejiang Academy of Agricultural Sciences, Hangzhou 310021, China; renhy@zaas.ac.cn (H.R.); hongywang1@gmail.com (H.W.); qixj@zaas.ac.cn (X.Q.); yuzp@zaas.ac.cn (Z.Y.); zhengxl@zaas.ac.cn (X.Z.); zhangsw@zaas.ac.cn (S.Z.); 2 School of Horticulture and Landscape architecture, Yangtze University, Jingzhou 434023, China; 3 College of Plant Protection, China Agricultural University, Beijing 100193, China; 4 Institute of Biotechnology, Zhejiang University, Hangzhou 310058, China; 11816060@zju.edu.cn (M.Z.); temoorahmed248@zju.edu.cn (T.A.)

**Keywords:** bayberry, decline disease, soil properties, microbial community, secondary metabolites

## Abstract

Decline disease causes serious damage and rapid death in bayberry, an important fruit tree in south China, but the cause of this disease remains unclear. The aim of this study was to investigate soil quality, microbial community structure and metabolites of rhizosphere soil samples from healthy and diseased trees. The results revealed a significant difference between healthy and diseased bayberry in soil properties, microbial community structure and metabolites. Indeed, the decline disease caused a 78.24% and 78.98% increase in *Rhizomicrobium* and *Cladophialophora*, but a 28.60%, 57.18%, 38.84% and 68.25% reduction in *Acidothermus*, *Mortierella*, *Trichoderma* and *Geminibasidium*, respectively, compared with healthy trees, based on 16S and ITS amplicon sequencing of soil microflora. Furthermore, redundancy discriminant analysis of microbial communities and soil properties indicated that the main variables of bacterial and fungal communities included pH, organic matter, magnesium, available phosphorus, nitrogen and calcium, which exhibited a greater influence in bacterial communities than in fungal communities. In addition, there was a high correlation between the changes in microbial community structure and secondary metabolites. Indeed, GC–MS metabolomics analysis showed that the healthy and diseased samples differed over six metabolic pathways, including thiamine metabolism, phenylalanine–tyrosine–tryptophan biosynthesis, valine–leucine–isoleucine biosynthesis, phenylalanine metabolism, fatty acid biosynthesis and fatty acid metabolism, where the diseased samples showed a 234.67% and 1007.80% increase in palatinitol and cytidine, respectively, and a 17.37–8.74% reduction in the other 40 metabolites compared to the healthy samples. Overall, these results revealed significant changes caused by decline disease in the chemical properties, microbiota and secondary metabolites of the rhizosphere soils, which provide new insights for understanding the cause of this bayberry disease.

## 1. Introduction

Bayberry (*Myrica rubra*) is an important fruit tree in southern China that is cultivated on approximately 334,000 hectares and has an annual output of approximately 950,000 tons. It is also an important Figmedicinal plant, and its extract contains antioxidants that can fight inflammation, allergies, diabetes, cancer, bacterial infection, and diarrhea [1,2]. It is well known that the main disease of bayberry is twig blight, which is mainly caused by *Pestalotiopsis versicolor* and *Pestalotiopsis microspora* [3]; the infected plants may die within one to four years. However, in recent years, bayberry plants have been infected by a new decline disease, with wide and serious effects, resulting in new shoots being difficult to pull, photosynthetic rate reduction, and trees dying 3–4 years later [4]. Until now, no pathogenic microbe has been successfully isolated from the infected bayberry plants, although a lot of work has been carried out on this disease.

Interestingly, Ren et al. (2021) [5] recently found that three kinds of bio-organic fertilizer have certain effects on decline disease in bayberry trees and could improve the content of nutrient elements in the soil and leaves of diseased trees, promoting vegetative growth and fruit quality. As we know, soil microbes play an important role in soil quality and fertility, and vice-versa [6]. Furthermore, soil metabolites have also been reported to be involved in interactions between microorganisms and plants by regulating numerous plant growth, development, and stress response processes [7]. Therefore, we proposed a hypothesis that this decline disease in bayberry may be associated with soil properties as well as soil microbial communities and metabolites.

The aim of this paper is to compare the differences between health and diseased bayberry in chemical properties, as well as microbial community structure and metabolites of rhizosphere soils. The results of this study will not only provide a scientific basis for understanding the cause of decline disease, but also give a microbial insight into the sustainable development of bayberry.

## 2. Materials and Methods

### 2.1. Investigated Orchard and Bayberry Trees

This study was carried out on fifteen-year-old bayberry (cv. Dongkui) trees, which were cultivated in Qianjiang Village (30°32′ N; 120 °42′ E) of Huangwan Town, Haining City, Zhejiang Province. The orchard was characterized by a typical gentle slope mountain area, approximately 50 m above sea level, and acidic yellow soil. Sixty-five percent of the bayberry plants in the orchard were infected by decline disease with 1–9 grades, which were ranked as described by Ren et al. (2020) [4]. The row spacing of bayberry plants was 4 m × 5 m, with bayberry trees with similar loads and crown sizes being selected for investigation and analysis in this study. The straight-line distance was about 100 m between the investigated healthy and decline bayberry trees with a disease index of grade 5. The orchard was managed conventionally.

### 2.2. Soil Sample Collection

Rhizosphere soil samples (0–20 cm) were collected in July 2016 at the drip line (1.5 m distance) around the crown of the healthy and diseased bayberry plants at the postharvest time of the fruits with stable vegetative growth and reproductive growth. Each treatment consists of 6 replicates and each replicate contains one tree. Using the quartering method, approximately 2 kg of mixed soil samples were collected from each investigated tree and passed through a 0.45 mm sieve. One half of the soil samples was stored in a refrigerator at −80 °C for DNA extraction, and the other half of the soil samples were dried at room temperature for measurement of soil properties.

### 2.3. Soil Genome Sequencing

Genomic DNA of soil samples was extracted by a DNA extraction kit (Foregene, Chengdu Fuji Biotechnology Co., Ltd., Chengdu, China). Bacterial diversity was determined by amplifying the 16S rRNA V3-V4 region using 343F-5′-TACGGAGGCAGAG-3′ and 798R-5′-AGGGTATCTATCT-3′ primers [8]. Fungal diversity was determined by amplifying the ITS1 and ITS2 region using ITS1F-5′-CTTGGTCATTTAGGAAGTAA-3′ and ITS2-5′-GCTGCGTTCTTCATTCGATGC-3′ primers. After purification of the PCR products with Agencourt AMPure XP beads (Beckman Coulter Co., Chaska, MN, USA) and quantification using a Qubit dsDNA assay kit, the amplicon quality was visualized using gel electrophoresis. The concentrations were then adjusted for genome sequencing, which were carried out by Shanghai Ouyi Biomedical Technology Co., Ltd. (Shanghai, China) using an Illumina MiSeqPE300 platform with two paired-end read cycles of 300 bases each [9].

Paired-end reads were preprocessed using Trimmomatic software [10] to cut off ambiguous bases and low-quality sequences with average quality scores below 20 using a sliding window trimming approach, and then assembled using FLASH software [11] with parameters of 10 bp minimal overlapping, 200 bp maximum overlapping and 20% maximum mismatch rate. Further denoising was achieved by removing the reads with chimeric, ambiguous, or homologous sequences or those below 200 bp. Following primer sequence removal, clean reads were subjected to clustering to generate operational taxonomic units (OTUs) using VSEARCH software with a 97% similarity cutoff [12]. After selection of the representative read of each OTU using the QIIME package [13], all 16S rDNA and ITS representative reads were annotated and blasted against the Silva database (Version 123) using the RDP classifier (confidence threshold was 70%) [14], and in the Unite database (ITSs rDNA) using BLAST [15], respectively. Alpha diversity was estimated using the Chao1 index [16] and Shannon index [17]. The unweighted unifrac distance matrix obtained by QIIME was used for principal coordinates analysis (PCoA) and phylogenetic tree construction.

### 2.4. Soil Properties

After natural air-drying, soil properties were examined. Indeed, the pH was determined by pH meter (the ratio of soil to water was 1:2.5); organic matter was determined by the K_2_Cr_2_O_7_ oxidation external heating method [18]; available N was determined by the Modified Kjeldahl method; available P was determined by hydrochloric acid–ammonium fluoride extraction molybdenum–antimony anti-colorimetry [19]; available calcium and magnesium were extracted by ammonium acetate, and contents were determined by an ice3500 atomic absorption spectrophotometer [20].

### 2.5. GC–MS Metabolomics Analysis

One gram of soil sample was added with internal standard (0.3 mg/mL L−2-chloro-phenylalanine dissolved in 20 μL methanol), 1 mL of methanol: water (*v*:*v* = 1:1), and two small steel balls. After precooling at −20 °C for 2 min and grinding with a grinder (60 Hz, 2 min), The homogenized sample was transferred to a 15 mL centrifuge tube, and the residue on the tube wall was repeatedly washed with 1 mL of methanol: water (*v*:*v* = 1:1). After centrifuging the total 3 mL sample solutions at 7700 rpm under 4 °C for 10 min, 2.5 mL of supernatant was placed into a 5 mL centrifuge tube and freeze-dried, and the dried powder was re-dissolved with 400 μL of methanol: water (*v*:*v* = 1:1), vortexed for 60 s and ultrasonicated for 30 s. The solution was transferred to a 1.5 mL centrifuge tube and centrifuged for 10 min (12,000 rpm, 4 °C), and 300 μL of supernatant was placed into a glass derivative bottle. The quality control sample (QC) was prepared by mixing the extract of all samples in equal volumes, and the volume of each QC sample was the same as that of the tested sample. The samples were dried in a freeze-dryer concentration centrifuge. To each glass derivation flask was added 80 μL of pyridine methoxyamine hydrochloride solution (15 mg/mL), and an oximation reaction was carried out in a shaking incubator at 37 °C for 90 min after vortex shaking for 2 min. The samples then had80 μL of BSTFA (containing 1% TMCS) derivatization reagent and 20 μL of n-hexane added. After vortexing for 2 min and reacting at 70 °C for 60 min, the samples were stored at room temperature for 30 min for gas chromatography–mass spectrometry (GC–MS) metabolomics analysis.

Metabolomics analysis was carried out on a 7890B-5977A GC/MSD GC–MS (Agilent Technologies Inc. Santa Clara, CA, USA) with a DB-5MS capillary column (30 m × 0.25 mm × 0.25 μm, Agilent J & W scientific, Folsom, CA, USA) and high-purity helium (purity no less than 99.999%). The flow rate was 1.0 mL/min, while the injection temperature was 260 °C. The injection volume was 1 μL and the solvent was delayed for 5 min. Temperature programming was as follows: the initial temperature of the column incubator was 60 ℃, followed by 8 °C/min to 125 ℃, 4 °C/min to 210 ℃, 5 °C/min to 270 °C and 10 °C/min to 305 °C for 3 min. The MS ion source and quadrupole temperature was 230 ℃ and 150 °C, respectively, while electron energy was 70 eV and scanning range was 50–500 *m*/*z*. The repeatability of the entire analysis process was examined by inserting one QC sample among every 10 samples. The obtained data in this study were compared with the standard spectrum library of the National Institute of Standards and Technology, and the obtained metabolite information was searched for in the KEGG database.

### 2.6. Statistical Analysis

PCoA, community histograms and redundancy discriminant analysis (RDA) were performed using R 3.5.1. A heat map was drawn by Pheatmap software. Excel 2010 was used for preliminary data processing and SPSS 17.0 software was used for the significance test (*p* < 0.05). The Kruskal–Wallis test was used to compare α-diversity metrics.

## 3. Results and Discussion

### 3.1. Distribution of Diseased Bayberry Trees

**Figure 1:**
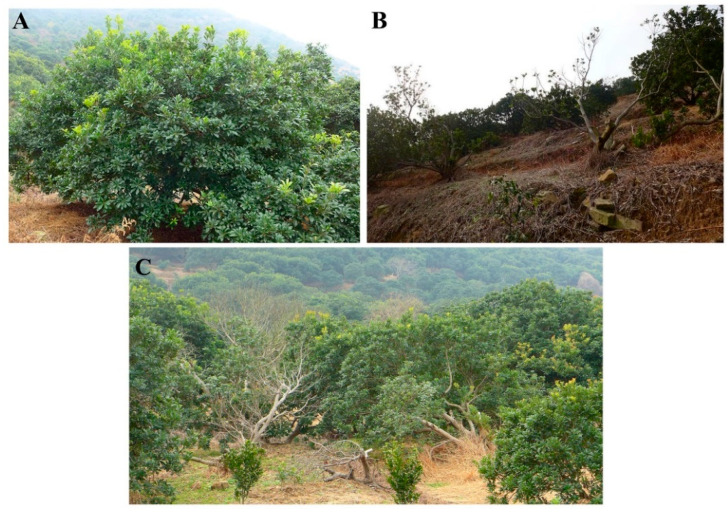
Bayberry trees orchard investigated in this study. Healthy (**A**) and diseased (**B**) bayberry trees and the distribution (**C**) of healthy and diseased bayberry trees.

The results of our investigation indicated that the locations of the diseased bayberry trees were highly random, with no center for the decline disease in the same orchard, although the disease worsens year by year until death, leading to soil quality being proposed to be one of the main causes of the disease. Furthermore, there was no difference in disease occurrence among different locations including the foot, middle or top of the mountain, with no obvious disease transmission trend in the investigated orchard (Figure 1). This result is consistent with the data of our previous reports [4]. In contrast, our recent studies found that the occurrence of twig blight caused by fungal infection is more serious at the foot of the mountain than that at the top of the mountain in the same orchard, and there is an obvious disease center and a trend of disease transmission. In addition, the incidence of twig blight was affected by temperature and relative humidity [4].

### 3.2. Changes in Microbial Community Diversity

As shown in Table 1, there was a difference in the number of operational taxonomic units (OTUs) in the bacterial V3 + V4 region and fungal ITS region between the healthy and diseased bayberry plants. Indeed, the average number of bacterial OTUs in healthy and diseased bayberry trees is 1685.50 (varying from 1480 to 1845) and 1575.33 (varying from 1203 to 1781), respectively (Table 1)—indicating that the diseased bayberry trees caused a 6.54% reduction in the number of bacterial OTUs of rhizosphere soils compared to those of healthy bayberry trees. The average number of fungal OTUs in healthy and diseased bayberry trees is 909.67 (varying from 736 to 1126) and 814.17 (varying from 725 to 884), respectively (Table 1)—suggesting that the diseased bayberry trees caused a 10.50% reduction in the number of fungal OTUs of rhizosphere soils compared to that of healthy bayberry trees.

Rhizosphere soil bacterial and fungal community diversity was determined in this study based on 16S rRNA and ITS amplicon sequencing analysis, respectively, as well as calculation of Chao1 and Shannon indexes after normalizing data from each sample to the same number of reads. As shown in Table 1, the bacterial and fungal Chao1 indexes of the diseased trees were 9.78% and 12.14% lower than those of healthy trees, while there was no significant (*p* < 0.05) difference in bacterial and fungal Shannon indexes between diseased and healthy trees. Furthermore, bacterial Chao1 indexes were 2.28-fold greater than those of fungi in healthy rhizosphere soil, while bacterial Chao1 indexes were 2.34-fold greater than those of fungi in diseased rhizosphere soil. Similarly, bacterial Shannon indexes were 1.41-fold greater than those of fungi in healthy rhizosphere soil, while bacterial Shannon indexes were 1.28-fold greater than those of fungi in diseased rhizosphere soil.

The results of this study indicated that both bacterial and fungal diversity were lower in rhizosphere soil from diseased bayberry plants than those of healthy bayberry plants (Table 1). However, the total number of soil fungal OUTs exhibited a greater reduction than that of soil bacterial OTUs, suggesting that the soil fungi may be more sensitive to the soil environment of diseased bayberry trees. Furthermore, the total number of bacterial OTUs was in general higher than that of the total number of fungal OTUs, regardless of the healthy or diseased bayberry trees. Indeed, the total number of bacterial OTUs was 1.85-fold of the total number of fungal OTUs in healthy bayberry trees, while the total number of bacterial OTUs was 1.93-fold of the total number of fungal OTUs in diseased bayberry trees (Table 1).

In agreement with the results of this study, it has been proposed that soil microbial diversity may be able to serve as an indicator for the outbreak of plant disease, supporting the ecological significance of microbial communities in maintaining plant health. Indeed, Wu et al. [21] found that compared to those of healthy *Panax notoginseng*, microbial communities in rhizosphere soils of diseased plants showed a decrease in alpha diversity. Furthermore, previous studies also found that the improvement of rhizosphere microflora structure was able to increase cucumber, soybean and apple yields by improving the organic matter and total nitrogen content of plants [22,23,24]. However, different microorganisms played various beneficial, neutral and harmful functions in plant growth; therefore, it can be inferred that the rhizosphere microbial community structure will provide an effective theoretical base for understanding the cause of bayberry decline disease.

### 3.3. Changes in Soil Microbial Community Composition

**Figure 2:**
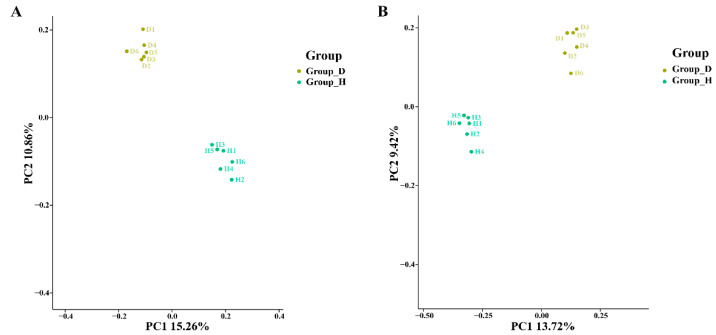
PCoA results from soil bacteria (**A**) and fungi (**B**) based on OTU abundance. H and D represent healthy tree and diseased trees, respectively.

In order to examine whether decline disease has an influence on the rhizosphere microbial community structure of bayberry plants, beta diversity analysis was performed to investigate the structural variation of microbial communities across samples using UniFrac distance metrics and visualized by PCoA. Indeed, the results of this study showed that the OTU abundance from six replicates of the healthy and diseased trees were clustered into two distinct groups and were well separated from each other regardless of soil fungi or bacteria, based on the PCoA analysis of unweight_unifrac distance (Figure 2, PERMANOVA; *p* < 0.05)—indicating that the microbial community composition varied between healthy and diseased trees.

**Figure 3:**
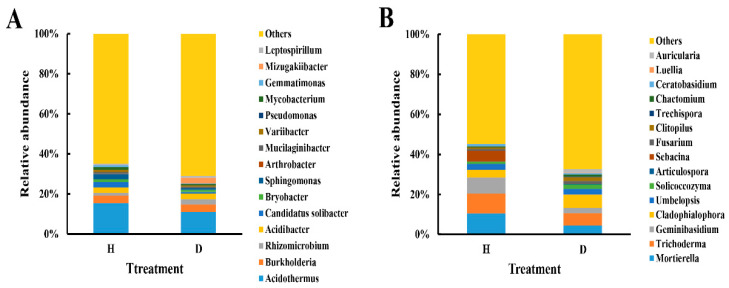
Relative abundance of bacteria (**A**) and fungi (**B**) at the genus level. H and D represent healthy and diseased trees, respectively.

This result indicated that there was a great difference in bacterial and fungal community composition at the phylum (Supplementary Figure S1), order (Supplementary Figure S2) and genus (Figure 3) levels between healthy and diseased bayberry trees, suggesting that compared to the healthy trees, the microbial community composition of the diseased bayberry rhizosphere soil was reconstructed. Among the 15 bacterial genera with a relative abundance greater than 1%, *Acidothermus*, *Burkholderia*, *Rhizomicrobium* and *Acidibacter* were the dominant genera (Figure 3A). Compared with the healthy trees, the relative abundance of *Acidothermus* was significantly (*p* < 0.05) decreased by 28.60%, and the relative abundance of *Rhizomicrobium* was significantly (*p* < 0.05) increased by 78.24% in the diseased trees. There was no significant change in other main bacterial genera. Notably, *Acidothermus cellulolyticus* produced two xylanases, which have the ability to efficiently use xylan, a major component of plant cell walls for conversion of plant biomass to products of interest [25]. Therefore, the decreased relative abundance of *Acidothermus* may reduce the biomass utilization ability of diseased bayberry plants.

Among the 15 fungal genera with a relative abundance greater than 1%, *Mortierella, Trichoderma, Geminibasidium* and *Cladophialophora* were the dominant genera, accounting for more than 30% of the fungal sequences (Figure 3B). Compared with the healthy bayberry trees, the relative abundances of *Mortierella*, *Trichoderma* and *Geminibasidium* in diseased bayberry trees were significantly (*p* < 0.05) decreased by 57.18%, 38.84% and 68.25%, respectively, while *Cladophialophora* in diseased trees was significantly (*p* < 0.05) increased by 78.98%. Interestingly, species of *Geminibasidium* are heat tolerant and xerotolerant, which makes this genus rich in soil. Furthermore, *Mortierella* is a kind of fungus that plays a beneficial role in plant growth promotion [26], while *Trichoderma* is an important biocontrol fungus that protects crops against pathogen infection [27].

### 3.4. Soil Properties Related to Microbial Communities

#### 3.4.1. Soil Properties

Results from this study indicated that there was a significant (*p* < 0.05) change in the pH, organic matter, magnesium, available nitrogen, phosphorus and calcium between healthy and diseased bayberry rhizosphere soil. Indeed, the pH, organic matter, magnesium, available nitrogen, phosphorus and calcium in healthy bayberry rhizosphere soil were 5.17, 4.38%, 53.58 mg/kg, 147.38 mg/kg, 16.64 mg/kg and 591.50 mg/kg, respectively. However, compared to the healthy bayberry, the decline disease resulted in a 9.28%, 36.07%, 33.67%, 20.00% and 41.11% reduction in pH, organic matter, magnesium, available nitrogen and calcium, respectively, but an 84.98% increase in available phosphorus (Table 2).

In agreement with the results of this study, previous studies also revealed that there was a difference between healthy and diseased trees with regards to pH value, organic matter content, available nitrogen, calcium, magnesium and phosphorus. Indeed, Ren et al. [4] reported that the pH, organic matter, and available nitrogen, calcium, magnesium, sulfur, zinc, copper, iron, manganese and boron of rhizosphere soil of diseased trees with disease degrees of grade 5 were significantly reduced, however, the contents of available phosphorus and potassium were significantly increased compared to the healthy trees. Interestingly, it is well known that most of these elements in rhizosphere soil are essential nutrients for plants over the whole growth period. In particular, more attention should be paid to the changes in the three heavy metals iron, zinc and copper, which have been reported to play fundamental roles in eucaryotes and procaryotes, and as their bioavailability regulates host–pathogen interactions [28].

On the other hand, it has been well documented that the growth of soil microbes is affected by a series of environmental factors such as soil pH, organic matter and magnesium content, as well as available nitrogen, phosphorus and calcium [29,30]. Furthermore, the results of this study revealed the complexity of the relationship between microbial growth and soil nutrient elements due to the fact that a differential change was observed between diseased and healthy bayberry rhizosphere soil in the contents of available soil nutrient elements, such as available nitrogen, calcium and phosphorus, as well as the magnesium content; indeed, nutritional balance may be best for microbial growth. However, the pH and organic matter have been proposed to exhibit a greater effect on microbial communities compared to the other soil parameters [31,32,33]. Therefore, the decrease in soil pH and organic matter in this study are expected to have a negative influence on microbial growth and diversity of diseased bayberry rhizosphere soil.

As we know, bacteria of the *Acidothermus* genus were able to grow under acidic conditions and degrade plant tissues [25,34], which normally cause increases in organic matter and magnesium content, as well as available nitrogen and calcium. Interestingly, most of these soil parameters are associated with soil pH [35]. Thus, it can be inferred that the lower pH in diseased bayberry rhizosphere soil may be partially due to the reduction in the relative abundance of *Acidothermus*. Similarly, the relative abundances of *Mortierella* and *Trichoderma* are higher in healthy than in diseased bayberry rhizosphere soil, while fungi from the two genera can not only promote plant growth [26,27], but also degrade lignin and cellulose [36,37], which results in increases in organic matter and magnesium content, as well as available nitrogen, calcium and phosphorus. However, bacteria from the genus *Rhizomicrobium* have been reported to participate in phosphate and phosphite metabolism in soil [38]. Therefore, it can be speculated that the increase in available phosphorus in diseased bayberry rhizosphere soil may be due to the fact that *Rhizomicrobium* has a greater effect on available phosphorus compared to *Acidothermus*, *Mortierella* and *Trichoderma*.

#### 3.4.2. RDA of Soil Properties and Microbial Communities

**Figure 4:**
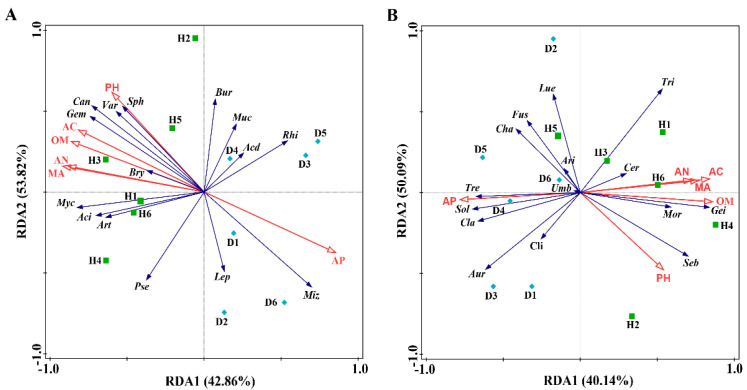
Redundancy discriminant analysis (RDA) of the rhizosphere bacterial (**A**) and fungal (**B**) community compositions at genus levels with soil physicochemical properties. Aci: *Acidothermus*; Bur: *Burkholderia*; Rhi: *Rhizomicrobium*; Acd: *Acidibacter*; Can: *Candidatus Solibacter*; Bry: *Bryobacter*; Sph: *Sphingomonas*; Art: *Arthrobacter*; Muc: *Mucilaginibacter*; Var: *Variibacter*; Pse: *Pseudomonas*; Myc: *Mycobacterium*; Gem: *Gemmatimonas; Miz*: *Mizugakiibacter*; Lep: *Leptospirillum*; Mor: *Mortierella*; Tri: *Trichoderma*; Gei: *Geminibasidium*; Cla: *Cladophialophora*; Umb: *Umbelopsis*; Sol: *Solicoccozyma*; Ari: *Articulospora*; Seb: *Sebacina*; Fus: *Fusarium*; Cli: *Clitopilus*; Tre: *Trechispora*; Cha: *Chaetomium*; Cer: *Ceratobasidium*; Lue: *Luellia*; Aur: *Auricularia*; OM: organic matter; AN: available nitrogen; AP: available phosphorus; AC: available calcium; MA: Magnesium. Green squares: samples from healthy bayberry; Blue diamonds: samples from diseased bayberry.

In this study, the association of soil properties with rhizosphere microbial community composition was examined using a redundancy discriminant analysis (RDA), which has been used previously to explore the relationship between environmental factors and microbial communities. Results from this study showed that there was a total of 96.68% and 90.23% of the cumulative variance of the rhizosphere microbial community-factor correction at the bacterial (Figure 4A) and fungal (Figure 4B) genus level, respectively. For bacterial communities at the genus level, the contributions of the six main variables were 36.6% by magnesium, 33.9% by available nitrogen, 33.3% by organic matter, 33.1% by available phosphorus, 30.6% by available calcium and 19.5% by pH. For fungal communities at the genus level, the contributions of the six main variables were 30.1% by organic matter, 28.8% by magnesium, 25.3% by available phosphorus, 24.5% by available calcium, 22.6% by available nitrogen, and 14.9% by pH, respectively (Table 3). In general, the results of this study showed that the compositions of the bacterial and fungal communities in bayberry rhizosphere soil were significantly affected by different soil properties. Although both bacterial and fungal communities were affected by the six main variables, the six main variables exhibited a greater influence in bacterial communities than in fungal communities.

### 3.5. Change in Rhizosphere Soil Metabolomics

**Figure 5:**
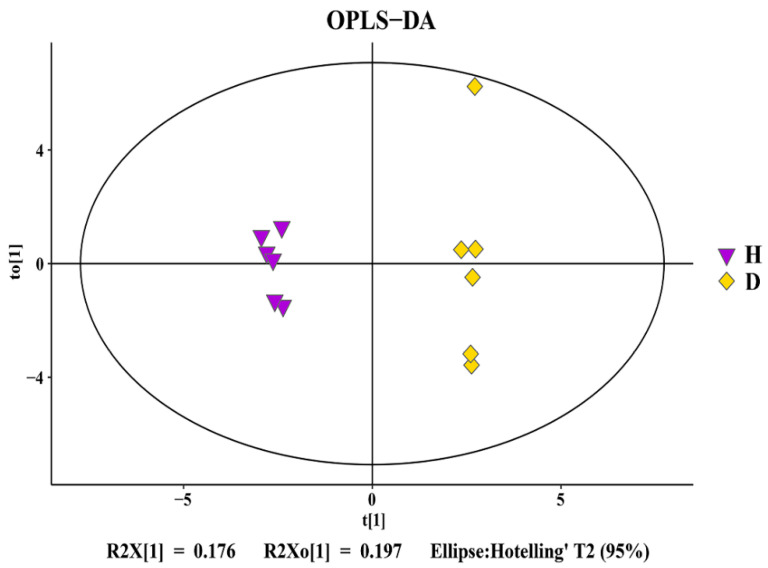
OPLS-DA score map of healthy (H) and diseased (D) bayberry rhizosphere soil.

A total of 223 rhizosphere soil metabolites were identified in this study using GC–MS analysis. Furthermore, a score map of metabolites was successfully constructed based on orthogonal partial least squares-discriminant analysis (OPLS-DA, a supervised statistical method of discriminant analysis), which can reduce intragroup differences, enlarge intergroup differences, and eliminate the influence of irrelevant factors on the experimental data to realize the effective prediction of different samples. Results showed that the distribution of healthy bayberry rhizosphere soils was well separated from that of diseased bayberry rhizosphere soils. Indeed, the healthy samples (H) were all distributed in the negative area of t[1] (principal component 1), while the decline disease samples (D) were all distributed in the positive area of t[1] (Figure 5). Furthermore, the parameters of the OPLS-DA are R^2^X(cum) = 0.425, R^2^Y(cum) = 0.974, R^2^ = 0.646, Q^2^(cum) = 0.237 (R^2^ > 0.5 and Q^2^ < 0.5), revealing the great interpretability and prediction ability of the H–D OPLS-DA model. These results suggests that the metabolite changes in bayberry rhizosphere soil are associated with decline disease.

### 3.6. Analysis of Differential Metabolites

**Figure 6:**
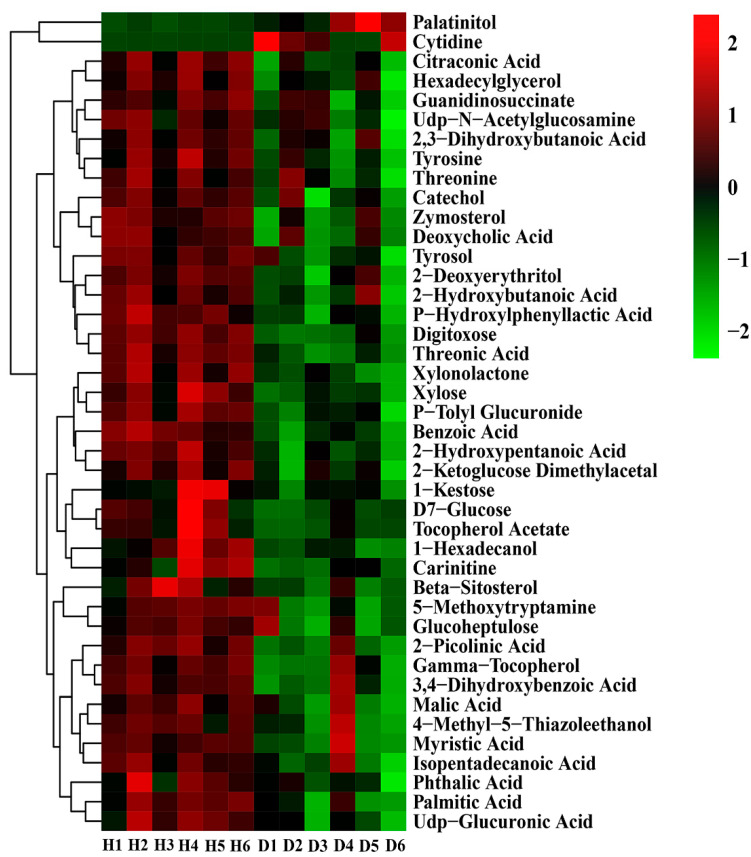
Thermogram analysis of different metabolites in bayberry rhizosphere soil. H and D represent healthy and diseased trees, respectively. The color scale is representative of the relative content of metabolites, where a high relative content is indicated in red and a low relative expression is indicated in green.

Amino acids, organic acids and other secondary metabolites are the biological sources of carbon and nitrogen for soil microbial growth and rhizosphere colonization, symbiosis and pathogenesis; they also play an important role in plant root growth, including regulating ion transport, participating in heavy metal detoxification and affecting the synthesis and activity of many important cellular enzymes [7,39]. In order to observe the change rules of metabolites, the metabolites with significant differences were normalized and the clustering heat map was drawn. Results indicated that the main intermediates were significantly changed between healthy and diseased bayberry rhizosphere soil (Figure 6, Table 4). Indeed, the contents of palatinitol and cytidine in diseased trees rhizosphere soil were 234.67% and 1007.80% higher than those of the healthy trees, respectively, suggesting that the two metabolites may be harmful to bayberry. In agreement with the results of this study, previous studies showed that some soil metabolites have toxic effects on plants [40,41] and change the richness of the bacterial community in rhizosphere soil [42].

Conversely, soil secondary metabolites are also beneficial to plants, contributing to changes in soil properties and regulation of rhizosphere soil microbial communities [43]. For example, phenolic compounds play important roles in weed growth inhibition by obtaining nutrients, allelopathy, regulating pH and enzyme activity, and participating in scavenging processes of plant reactive oxygen species and other ecological processes [44,45]. The increases in phenol and flavonoid metabolites can regulate nitrogen fixation and promote the growth of beneficial bacteria in maize rhizosphere soil [46]. In this study, the contents of the other 40 metabolites were 17.37–68.74% lower than those of the healthy trees (Figure 6, Table 4). Indeed, the relative contents of sugars (D7 glucose, xylonolactone, xylose, etc.) and organic acids (malic acid, citric acid) in diseased bayberry rhizosphere soil decreased significantly compared to the healthy bayberry rhizosphere soil. Furthermore, a significant reduction was also found in the content of tyrosine, which can regulate plant root tips and maintain root cells [43,47].

### 3.7. Analysis of Differential Metabolic Pathways

**Figure 7:**
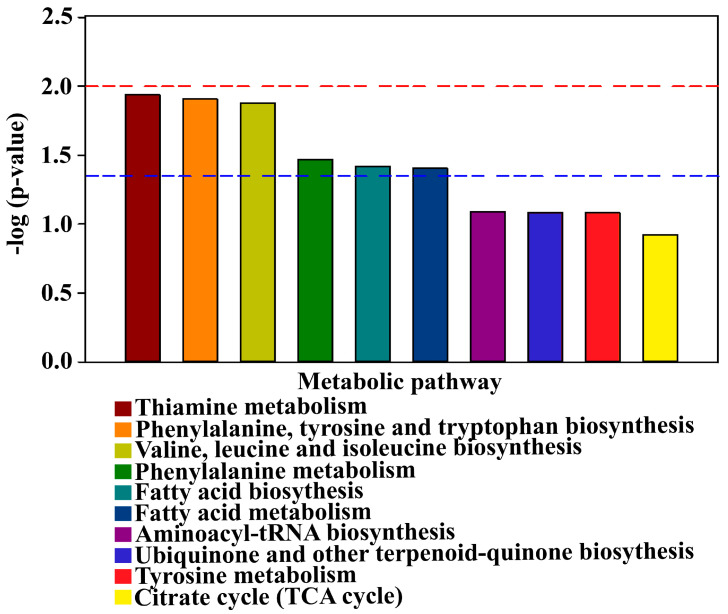
Metabolic pathway enrichment map of different metabolites in healthy (H) and diseased (D) bayberry rhizosphere soil. When the top of the bar is higher than the blue line or red line, the signal pathway it represents is significant.

The pathway enrichment analysis of different metabolites in healthy and diseased trees was conducted with the KEGG (Kyoto Encyclopedia of Genes and Genomes) database. Results showed that there was a significant difference between healthy (H) and diseased (D) trees in six metabolic pathways, including thiamine metabolism, phenylalanine–tyrosine–tryptophan biosynthesis, valine–leucine–isoleucine biosynthesis, phenylalanine metabolism, fatty acid biosynthesis and fatty acid metabolism (Figure 7), while there no significant difference between healthy and diseased trees in four other metabolic pathways including aminoacyl-tRNA biosynthesis, ubiquinone and other terpenoid–quinone biosynthesis, tyrosine metabolism and the citrate cycle. In agreement with the results of this study, phenylalanine, tyrosine and tryptophan are three aromatic amino acids involved in the synthesis of a variety of proteins, plant hormones, biopolymers and secondary metabolites. Tyrosine and phenylalanine are precursors of phenylalanine, alkaloids and glucosinolates, and their upregulated expression in whole-plant tissue indicates the activation of its defense system [48].

### 3.8. Correlation Analysis of Soil Microorganisms and Metabolites

**Figure 8:**
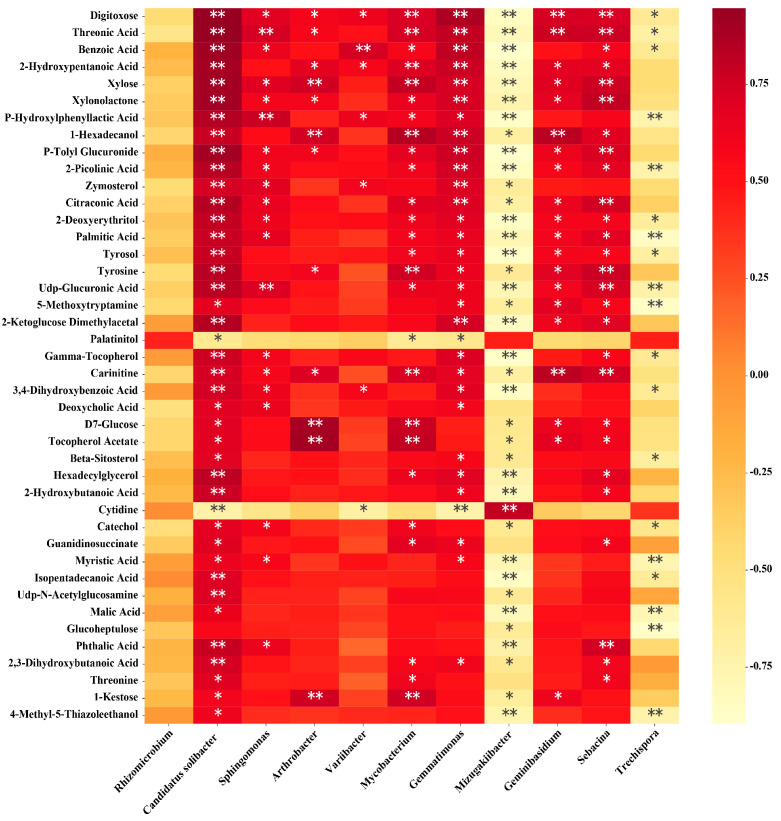
Heatmap of correlation analysis between microorganism relative abundances at genus level and metabolite relative contents. * and ** indicate a significant correlation at *p* < 0.05 and *p* < 0.01, respectively. The depth of the orange scale represents the magnitude of the correlation coefficient, where the darker the color, the greater the positive correlation.

This result revealed a correlation at the phylum, order and genus levels between microbial groups and secondary metabolites of bayberry rhizosphere soil (Figure 8, Supplementary Figures S3 and S4). At the genus level, digitoxose and threonic acid were positively correlated with *Mycobacterium*, *Geminibasidium* and *Sebacina*. There was a positive correlation between 1-hexadecanol and *Arthrobacter*,*Mycobacterium* and *Geminibasidium*. Cytidine and palatinitol were negatively correlated with *Gemmatimonas* and *Candidatus solibacter*, but positively correlated with *Mizugakiibacter*. The other 40 metabolites were positively and negatively correlated with *Gemmatimonas* and *Mizugakiibacter*, respectively. The other 40 metabolites, except glucoheptulose, were positively correlated with *Candidatus solibacter* (Figure 8).

On the other hand, at the genus level, *Sphingomonas* was positively correlated with threonic acid, p-hydroxylphenyllactic acid and UDP-glucuronic acid. *Arthrobacter* was positively correlated with xylose, D7-glucose, tocopherol acetate and 1-kestose. *Variibacter* was positively correlated with benzoic acid. *Mycobacterium* was positively correlated with 2-hydroxypentanoic acid, xylose, tyrosine, carnitine, D7-glucose, tocopherol acetate and 1-kestose. *Geminibasidium* was positively correlated with carnitine. *Sebacina* was positively correlated with xylose, xylonolactone, P-hydroxylphenyllactic acid, citraconic acid, tyrosine, UDP-glucuronic acid, carnitine and phthalic acid. *Trechispora* was negatively correlated with P-hydroxylphenyllacticacid, 2-picolinic acid, palmitic acid, UDP-glucuronic acid, 5-methoxytryptamine, myristic acid, malic acid, glucoheptulose and 4-methyl-5-thiazoleethanol. *Gemmatimonas* and *Mizugakiibacter* are likely to be the core microbes mediating microorganisms and metabolites (Figure 8).

## 4. Conclusions

The results of this study indicated that there was a significant difference in bayberry rhizosphere soil microbiota between healthy and diseased samples. Indeed, the relative abundances of *Rhizomicrobium* and *Cladophialophora* in diseased trees was greater than that of healthy trees, while the relative abundances of *Acidothermus, Mortierella*, *Trichoderma* and *Geminibasidium* in diseased trees was less than that of healthy trees. Furthermore, RDA of microbial communities and soil properties indicated that pH, organic matter, magnesium, available phosphorus, nitrogen and calcium are the main variables of bacterial and fungal communities, where the influence of these six main variables in bacterial communities was greater than that in fungal communities. In addition, changes in microbial community structure may be able to be supported by high correlations between microorganisms at the phylum, order and genus levels and secondary metabolites. Indeed, GC–MS metabolomics analysis of bayberry rhizosphere soil showed that the healthy and decline disease trees differed over six metabolic pathways, including thiamine metabolism, phenylalanine–tyrosine–tryptophan biosynthesis, valine–leucine–isoleucine biosynthesis, phenylalanine metabolism, fatty acid biosynthesis and fatty acid metabolism, where the contents of palatinitol and cytidine were higher in the rhizosphere soil of diseased trees than those of the healthy trees, but the contents of the other 40 different metabolites, including sugars, organic acids and secondary metabolites, were lower than those of healthy trees. Overall, the results of this study revealed significant changes between healthy and diseased trees in microbiota, chemical properties, and secondary metabolites of their rhizosphere soils, which provides us with a new way to explore the cause of this bayberry disease.

## Figures and Tables

**Table 1:** Diversity indexes and OTU distribution of bacteria and fungi in healthy and decline disease bayberry rhizosphere soil.

Microorganism	Treatment	Chao1 Index	Shannon Index	OTUs
Bacteria	Healthy	2295.77 ± 174.80	8.54 ± 0.17	1685.50 ± 141.60
	Diseased	2071.23 ± 234.20 ^#^	7.86 ± 0.76	1575.33 ± 211.98
Fungi	Healthy	1005.80 ± 155.13	6.04 ± 0.50	909.67 ± 135.91
	Diseased	883.69 ± 68.78 ^#^	6.12 ± 0.28	814.17 ± 70.56

^#^ in the same column indicates significant (*p* < 0.05) differences between healthy and diseased bayberry within either bacterial or fungal microbiota.

**Table 2:** The pH and chemical properties of healthy and diseased bayberry rhizosphere soil.

Parameters	Value	Parameters	Value
pH		Available phosphorus (mg/kg)	
H	5.17 ± 0.23	H	16.64 ± 0.50
D	4.69 ± 0.39 ^#^	D	30.78 ± 0.50 *
Organic matter (%)		Available calcium (mg/kg)	
H	4.38 ± 0.13	H	591.50 ± 20.17
D	2.80 ± 0.12 ^#^	D	348.33 ± 9.49 ^#^
Available nitrogen (mg/kg)		Magnesium (mg/kg)	
H	147.38 ± 6.01	H	53.58 ± 3.00
D	117.90 ± 4.58 ^#^	D	35.54 ± 1.12 ^#^

H and D represent healthy and diseased trees, respectively. The * and ^#^ represent significantly increases or decreases compared to healthy trees (*p* < 0.05).

**Table 3:** Contribution of soil environment to bacteria and fungi taxa at the genus level.

Taxa	Soil Environment	Contribution (%)
Bacteria		
	pH	19.5
	Organic Matter	33.3
	Available Nitrogen	33.9
	Available Phosphorus	33.1
	Available Calcium	30.6
	Magnesium	36.6
Fungi		
	pH	14.9
	Organic Matter	30.1
	Available Nitrogen	22.6
	Available Phosphorus	25.3
	Available Calcium	24.5
	Magnesium	28.8

**Table 4:** The relative contents of the differential metabolites in rhizosphere soil between healthy and decline disease trees.

Metabolite Name	Relative Content	Metabolite Name	Relative Content
Palatinitol		Benzoic Acid	
H	1.03 ± 0.12	H	3.69 ± 0.28
D	3.44 ± 0.79 *	D	2.45 ± 0.40 ^#^
Cytidine		2-Hydroxypentanoic Acid	
H	1.66 ± 0.15	H	1.96 ± 0.23
D	18.40 ± 3.39 *	D	1.51 ± 0.16 ^#^
Citraconic Acid		2-Ketoglucose Dimethylacetal	
H	7.05 ± 0.67	H	0.23 ± 0.03
D	5.12 ± 0.51 ^#^	D	0.16 ± 0.05 ^#^
Hexadecyl glycerol		1-Kestose	
H	5.89 ± 0.75	H	52.15 ± 4.21
D	3.87 ± 0.40 ^#^	D	24.28 ± 2.97 ^#^
Guanidinosuccinate		D7-Glucose	
H	0.50 ± 0.05	H	2.29 ± 1.00
D	0.36 ± 0.02 ^#^	D	0.87 ± 0.21 ^#^
Udp-N-Acetylglucosamine		Tocopherol Acetate	
H	0.98 ± 0.09	H	3.84 ± 0.89
D	0.77 ± 0.07 ^#^	D	1.20 ± 0.16 ^#^
2,3-Dihydroxybutanoic Acid		1-Hexadecanol	
H	1.28 ± 0.08	H	6.59 ± 1.11
D	1.06 ± 0.10 ^#^	D	4.22 ± 0.61 ^#^
Tyrosine		Carnitine	
H	2.70 ± 0.32	H	9.95 ± 1.73
D	1.91 ± 0.12 ^#^	D	4.33 ± 0.97 ^#^
Threonine		Beta-Sitosterol	
H	2.21 ± 0.24	H	2.64 ± 0.65
D	1.68 ± 0.17 ^#^	D	1.64 ± 0.12 ^#^
Catechol		5-Methoxytryptamine	
H	0.37 ± 0.02	H	57.95 ± 5.44
D	0.28 ± 0.08 ^#^	D	38.13 ± 2.79 ^#^
Zymosterol		Glucoheptulose	
H	0.85 ± 0.09	H	5.51 ± 0.35
D	0.46 ± 0.03 ^#^	D	3.90 ± 0.51 ^#^
Deoxycholic Acid		2-Picolinic Acid	
H	0.76 ± 0.09	H	3.03 ± 0.28
D	0.45 ± 0.01 ^#^	D	1.81 ± 0.10 ^#^
Tyrosol		Gamma-Tocopherol	
H	0.66 ± 0.05	H	1.88 ± 0.17
D	0.45 ± 0.03 ^#^	D	1.09 ± 0.17 ^#^
2-Deoxyerythritol		3,4-Dihydroxybenzoic Acid	
H	2.48 ± 0.17	H	9.86 ± 0.82
D	1.52 ± 0.18 ^#^	D	5.52 ± 0.18 ^#^
2-Hydroxybutanoic Acid		Malic Acid	
H	2.07 ± 0.14	H	1.65 ± 0.12
D	1.11 ± 0.13 ^#^	D	1.26 ± 0.15 ^#^
P-Hydroxylphenyllactic Acid		4-Methyl-5-Thiazoleethanol	
H	1.38 ± 0.18	H	0.36 ± 0.03
D	0.69 ± 0.09 ^#^	D	0.28 ± 0.07 ^#^
Digitoxose		Myristic Acid	
H	6.62 ± 0.48	H	3.14 ± 0.15
D	3.30 ± 0.29 ^#^	D	2.19 ± 0.23 ^#^
Threonic Acid		Isopentadecanoic Acid	
H	1.45 ± 0.12	H	0.51 ± 0.04
D	0.86 ± 0.04 ^#^	D	0.38 ± 0.01 ^#^
Xylonolactone		Phthalic Acid	
H	1.68 ± 0.14	H	2.14 ± 0.29
D	1.28 ± 0.14 ^#^	D	1.70 ± 0.30 ^#^
Xylose		Palmitic Acid	
H	1.16 ± 0.14	H	13.90 ± 1.03
D	0.79 ± 0.10 ^#^	D	10.39 ± 2.02 ^#^
P-Tolyl Glucuronide		Udp-Glucuronic Acid	
H	0.48 ± 0.05	H	0.41 ± 0.04
D	0.31 ± 0.08 ^#^	D	0.32 ± 0.05 ^#^

H and D represented healthy trees and diseased trees, respectively. ^“^*^”^ and ^“#”^ represent significantly increases or decreases compared to healthy tress (*p* < 0.05).

## Data Availability

Access to Sequence Read Archive metadata can be found with the following references: PRJNA720262 and PRJNA720289.

## Supplementary Materials

The following are available online at https://www.mdpi.com/article/10.3390/plants10102083/s1, Figure S1. Relative abundance of bacteria (A) and fungi (B) at the phylum level. H and D represent healthy tree and diseased trees, respectively; Figure S2. Relative abundance of bacteria (A) and fungi (B) at the order level. H and D represent healthy tree and diseased trees, respectively; Figure S3. Heatmap of correlation analysis between microorganism relative abundances at phylum level and the metabolite relative contents. * and ** indicate a significant correlation at *p* < 0.05 and *p* < 0.01, respectively; Figure S4. Heatmap of correlation analysis between microorganism relative abundances at order level and metabolite relative contents. * and ** indicate a significant correlation at *p* < 0.05 and *p* < 0.01, respectively.
