# Supplementary material for: The Damage Caused by Decline Disease in Bayberry Plants through Changes in Soil Properties, Rhizosphere Microbial Community Structure and Metabolites
Source: Plants (Basel). 2021 Sep 30;10(10):2083. doi: 10.3390/plants10102083 (PMC8540645; doi:10.3390/plants10102083)
Supplement: Supplementary file 1 [file plants-10-02083-s001.zip › plants-1350310-supplementary.pdf]

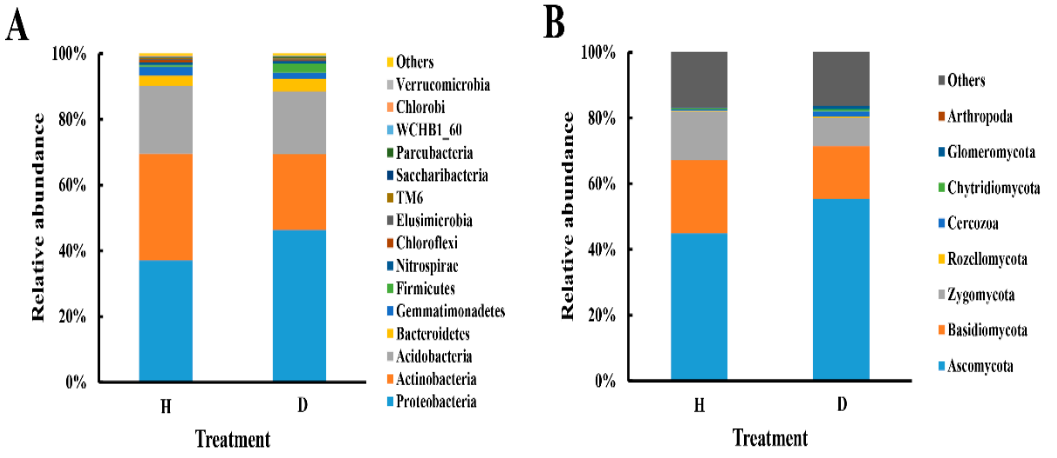

Figure S1. Relative abundance of bacteria (**A**) and fungi (**B**) at the phylum level. H and D represented healthy tree and diseased trees, respectively.

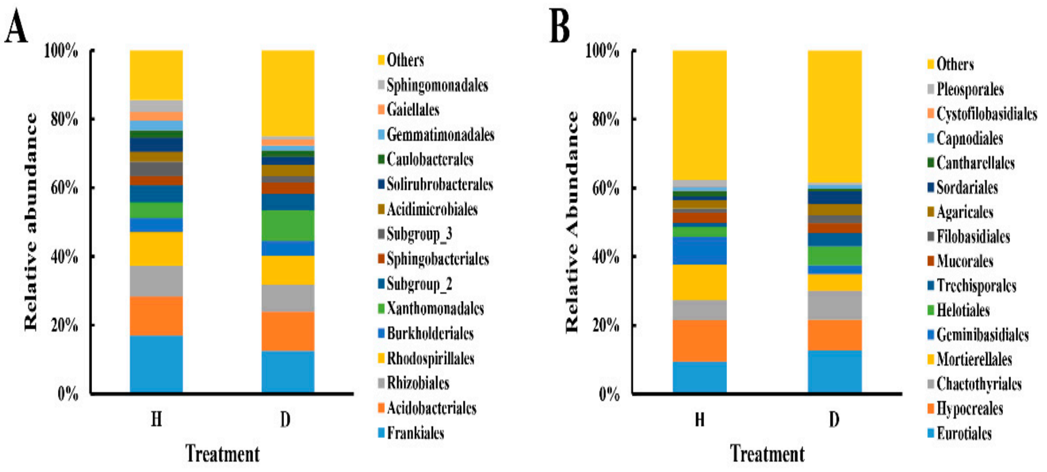

Figure S2. Relative abundance of bacteria (A) and fungi (B) at the order level. H and D represented healthy tree and diseased trees, respectively.

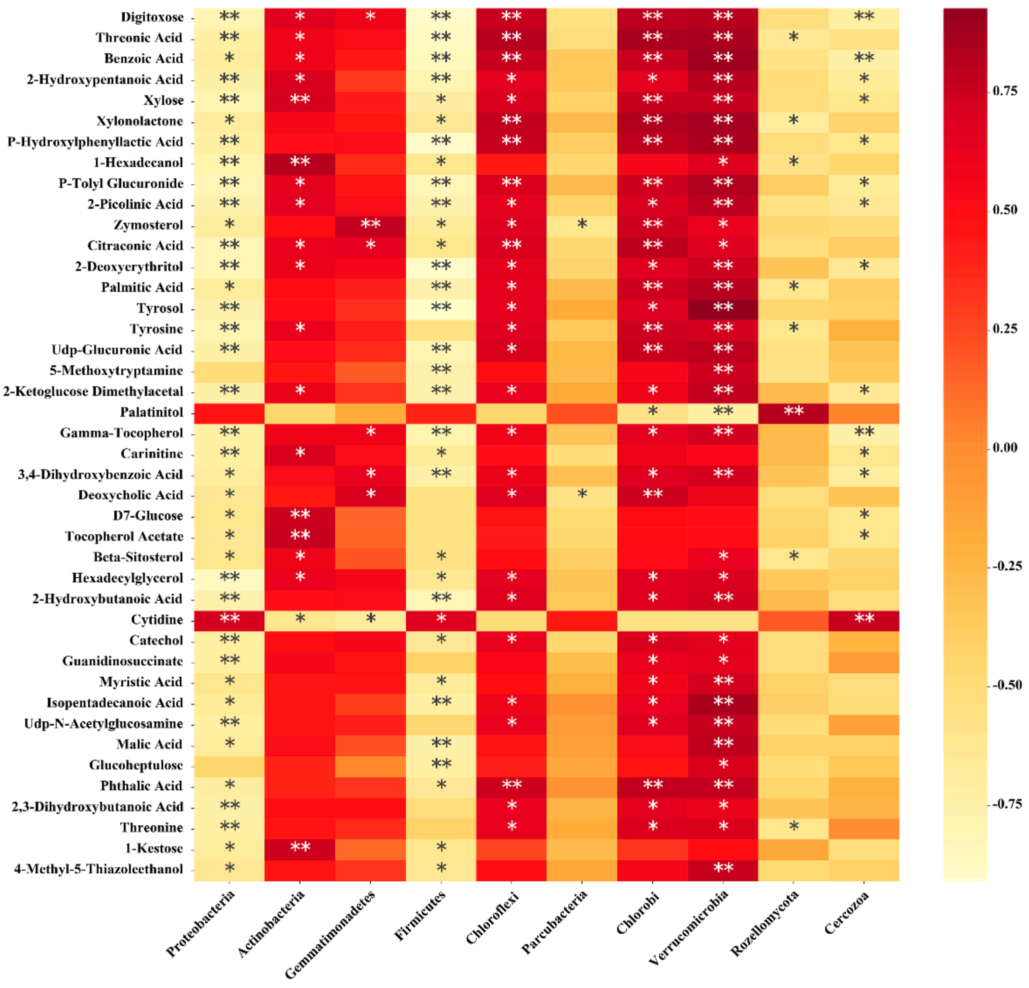

Figure S3. Heatmap of correlation analysis between the microorganism relative abundances at phylum level and the metabolite relative contents. * and ** indicate a significant correlation at $P < 0.05$ and $P < 0.01$, respectively.

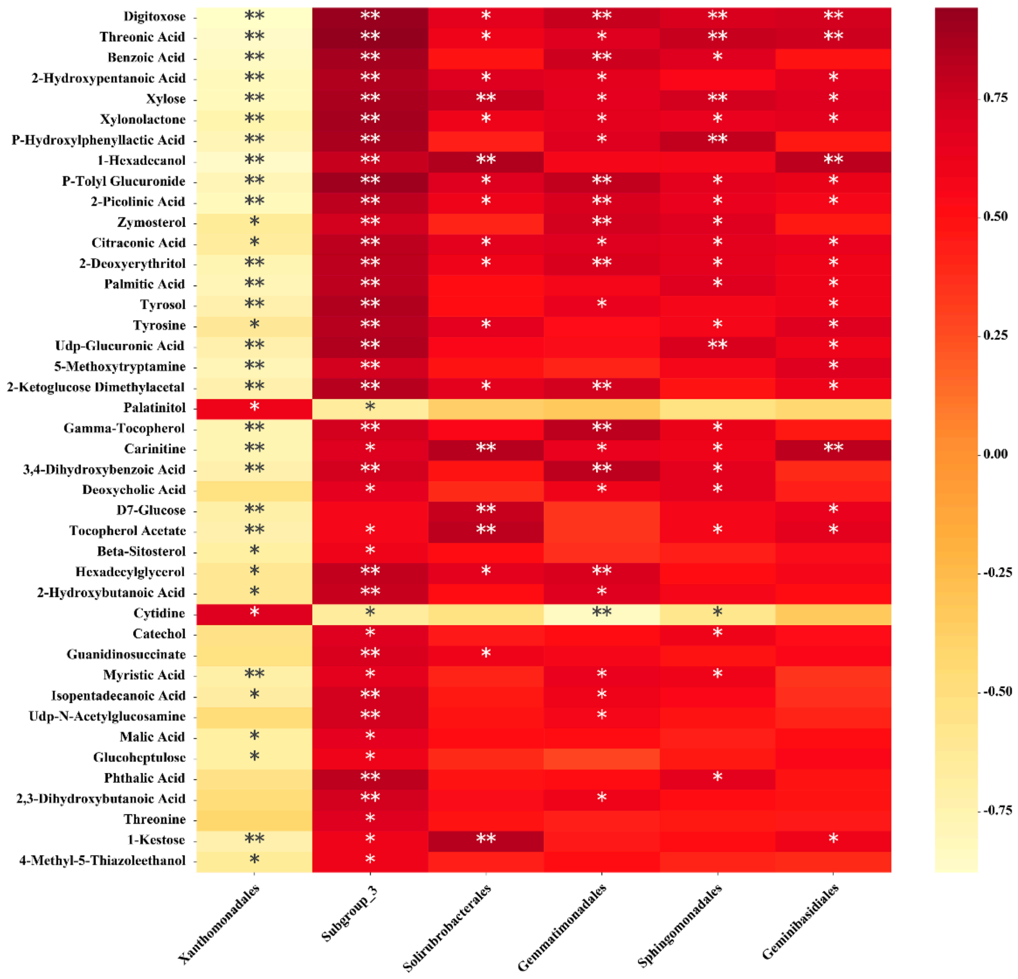

Figure S4. Heatmap of correlation analysis between the microorganism relative abundances at order level and the metabolite relative contents. * and ** indicate a significant correlation at $P < 0.05$ and $P < 0.01$, respectively.
